# Differential Gastric Motility, Gut Hormone, and Appetite Changes Following A Mixed Meal in People With Obesity and Healthy Controls

**DOI:** 10.1111/nmo.70163

**Published:** 2025-09-17

**Authors:** Darius Javidi, Dominic‐Luc Webb, Hetzel Olenia Diaz, Moeen Ud‐din, Khalid Elias, Magnus Sundbom, Per M. Hellström

**Affiliations:** ^1^ Department of Medical Sciences Uppsala University Uppsala Sweden; ^2^ Department of Medicine, Dartmouth‐Hitchcock Medical Center Lebanon New Hampshire USA; ^3^ Department of Surgical Sciences Uppsala University Uppsala Sweden

## Abstract

**Introduction:**

Understanding meal‐induced changes of gut hormones, gastric motility, and appetite is crucial for developing therapies for obesity. We investigated glucose‐dependent insulinotropic peptide (GIP), glucagon‐like peptide‐1 (GLP‐1), ghrelin, and motilin influences on gastric motility and appetite, to compare healthy individuals and people with obesity.

**Methods:**

Subjects (healthy *n* = 41; obesity *n* = 32) consumed a 270‐kcal meal and a wireless motility capsule. GIP, active GLP‐1, acyl‐ghrelin, and motilin were measured by electrochemiluminescence. MotiliGI and GIMS software were used for motility analysis, while visual analog scoring measured appetite.

**Results:**

Gastric emptying was more rapid in people with obesity than in healthy individuals (*p* < 0.01). Gastric emptying time was negatively associated with motility index and hunger contractions (*p* < 0.01, *p* < 0.05) in healthy individuals, but not in individuals with obesity. In controls, gastric motility index correlated positively with acyl‐ghrelin (*p* < 0.01) and motilin (*p* < 0.0001), and negatively with GIP (*p* < 0.05), but not aGLP‐1. In people with obesity, no gut hormones correlated with the motility index. In both groups, GIP and aGLP‐1 correlated with decreased hunger (*p* < 0.0001, *p* = 0.001) and (*p* < 0.0001, *p* < 0.05), along with increased satiety in controls (*p* < 0.0001, *p* = 0.001) and people with obesity (*p* = 0.049, *p* = 0.01). Acyl‐ghrelin correlated positively with hunger (*p* < 0.0001) and negatively with satiety (*p* = 0.049) in controls, but not in obesity. Motilin was neither associated with hunger nor satiety.

**Conclusion:**

In the gastric phase, people with obesity show rapid gastric emptying with altered hormone and motility meal responses. In healthy individuals, GIP promotes satiety, and ghrelin and motilin promote hunger through actions on motility. Like GIP, GLP‐1 promotes satiety along with trending suppression of gastric motility.

## Graphical abstract

During the gastric emptying phase after food intake, people with obesity show rapid gastric emptying as compared to healthy subjects. They also display low gut hormone excursions and flatline gastric motility responses. In subjects with obesity, the relationships between gastric motility, GIP, and GLP‐1 versus hunger and satiety were preserved, but disrupted for gastric stimulation by ghrelin and motilin.

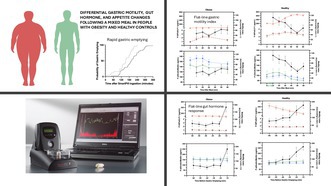

**Summary**

- People with obesity have more rapid gastric emptying. They are devoid of gastric motility response and have low hormone responses to a meal.
- In healthy, gastric motility correlates positively with acyl‐ghrelin and motilin, and negatively with GIP. In obesity these hormones do not correlate with motility.
- In both groups, GIP and GLP‐1 correlate with reduced hunger and increased satiety, while ghrelin, but not motilin, correlate positively with hunger and negatively with satiety in the healthy, but not in obesity.
- We conclude that the hormonal appetite interplay with gastric motility is disrupted in people with obesity.

## 1 Introduction

For the treatment of obesity, hybrid receptor ligands including both glucagon‐like peptide‐1 (GLP‐1) and glucose‐dependent insulinotropic peptide (GIP) have been shown to be particularly effective, surpassing the efficacy of GLP‐1 receptor single agonist medications [1]. Understanding the interplay between gastric motility, gut peptide hormones, and hunger/satiety during a mixed meal in both healthy individuals and individuals with obesity may offer important insights into the pathophysiology of obesity and aid in the development of new obesity pharmacotherapies.

GLP‐1 is an incretin whose plasma levels are lower in people with obesity than in healthy individuals, resulting in a blunted peak response to oral glucose [2, 3]. Infusion of GLP‐1 slows gastric emptying [4] and diminishes antral and duodenal motility in healthy volunteers and those with irritable bowel syndrome [5]. Exogenous administration of GLP‐1 dampens hunger, increases satiety, and prolongs the postprandial return of hunger contractions in both individuals with obesity [4] and those with normal body weight [6].

GIP is also an incretin that stimulates insulin secretion when glucose is high and glucagon secretion when glucose is low [7, 8]. Notably, in type 2 diabetes, the incretin effect of GIP is lost [9]. The effects of GIP on gastric function in humans have not been fully elucidated, but GIP has been shown to have a mild inhibitory effect on the secretion of gastric acid [10]. The role of GIP in weight loss is debated, where GIP receptor agonism, rather than antagonism, leads to weight loss in rodents [11]. However, paradoxical observations of GIP receptor agonists and antagonists appear to provide metabolic benefits when combined with GLP‐1 receptor agonists [12].

Ghrelin is an anti‐incretin that increases serum glucose and decreases insulin levels in healthy individuals [13], as well as individuals with obesity [14]. Ghrelin also increases food intake [15] and stimulates gastric emptying [16]. It is suggested to play an essential role in meal timing, with high ghrelin observed immediately before eating, and low ghrelin levels postprandially; counter‐regulatory to insulin [17].

Motilin is structurally similar to ghrelin, and their respective receptors are similar as well [18]. Administration of motilin leads to hastened gastric emptying [19], which is the mechanism by which it is proposed to increase hunger [20].

Studies investigating gastric motility in individuals with obesity have primarily focused on gastric emptying time (GET) as measured by gastric scintigraphy, with mixed conclusions [21, 22]. The relationship between the GET as measured by scintigraphy and appetite has also been investigated, with more rapid GETs associated with increased hunger [23]. Studies have also assessed how peripheral infusion of gut hormones affects gastric emptying [16, 24, 25, 26]. To our knowledge, no studies have investigated how endogenously produced gut hormones interact and relate to intragastric pressures, gastric emptying, and appetite following a standardized mixed meal.

Our study focuses on the gastric emptying process, from the intake of a mixed meal together with the SmartPill motility capsule, through the emptying process until the motility capsule leaves the stomach. The endocrine interplay of the two incretins GLP‐1 and GIP, and the two anti‐incretins ghrelin and motilin, was related to the detailed study of different phases of the gastric emptying process, along with simultaneous appetite scores.

The primary objective of our study was to elucidate differences between normal subjects and people with obesity in terms of gastric motility. A secondary objective was to relate differences in gastric motility and appetite scorings to the plasma concentrations of incretins and anti‐incretins by using correlation analyses.

We hypothesized that significant differences in gastric motility (as measured by GET and gastric motility index), appetite (as measured by Visual Analog Scales [VAS]), and gut hormone concentrations are present in healthy individuals and individuals with obesity. Moreover, we hypothesized that differences in gastric motility and appetite were associated with differences in gut hormone concentrations.

## 2 Methods

### 2.1 Participants and Study Design

A total of 32 people with obesity; 18 males, age 38.4 ± 11.3 years, body mass index (BMI) 56.5 ± 5.1 kg/m^2^, and 41 healthy control subjects; 20 males, age 28.4 ± 12.8 years, BMI 22.6 ± 2.1 kg/m^2^ (*p* < 000.1), were included in the study. Among those with obesity, 12 had hypertension (*p* < 0.0001), 10 type 2 diabetes (*p* = 0.0001), seven osteoarthritis (*p* < 0.002), and three dyslipidemia (*p* = 0.045); none of which were diagnosed in the healthy controls. All subjects were on appropriate pharmacological treatment with an angiotensin receptor blocker for hypertension, and metformin for type 2 diabetes, on‐demand treatment with NSAIDs for osteoarthritis, and atorvastatin or rosuvastatin for dyslipidemia. Participants reported stable weight during the 3 months before examination.

After an overnight fast, healthy subjects and people with obesity consumed a standardized 270 kcal mixed meal consisting of two egg whites, buttered toast, and jelly (3% fat, 21% protein, 76% carbohydrate, ~3% of which was fiber; % dry weight). The contents of the meal correspond to the energy content and macronutrient composition of the SmartBar, a standardized meal designed to be used with the SmartPill wireless motility capsule. The nutrient composition of the standardized meal used was similar to that of the typical meal used in gastric scintigraphy studies. The mixed meal was high in carbohydrate content and comparable to a light carbohydrate‐dominant breakfast, which is common in Western countries.

After the meal, the wireless motility recording device, the SmartPill capsule (Medtronic Inc., Minneapolis, MN, USA) was ingested with 100 mL of water. Subjects were ambulatory but encouraged to sit during the recording period, which covered the time from ingestion of the SmartPill capsule until it entered into the duodenum, as read by a continuous elevation of the recorded pH to a neutral level. Details of the methods have been published elsewhere [27].

The SmartPill measures luminal temperature, pressure, and pH. Temperature is measured every 5 s, pressure is measured every 0.5 s, and pH every 2 s. The integrated software MotiliGI 3.1 with the SmartPill equipment (Medtronic Inc.) was used to calculate GET. The GIMS software (Medtronic Inc.), developed for research purposes, was employed to analyze additional SmartPill motility data. The gastric motility index was calculated as the natural logarithm of pressure by seconds per minute in 10‐min intervals according to Ouyang et al. [28].

Hunger contractions were defined as contractile pressures exceeding 50 mmHg in the stomach. The hunger contraction frequency for each individual was calculated by dividing the total number of hunger contractions by their GET.

Blood samples were drawn from an antecubital vein at −10, 0, 10, 20, 30, 40, 50, 60, 90, 120, and 180 min into the meal. Blood samples were drawn into cold 10‐mL EDTA vacutainer tubes. Samples were immediately centrifuged (1500*g*, 4°C, 10 min), and the plasma supernatants were stored at −20°C until analysis. All samples were analyzed in duplicate. Intra‐assay variability was obtained through a quality control plasma sample on all plates, and inter‐assay variability was calculated from duplicate samples as percent coefficient of variation (CV%). Inter‐assay and intra‐assay coefficients of variation were: Active GLP‐1 10.8% and 26.6%, GIP 5.2% and 12.8%, acyl‐ghrelin 7.7% and 13.1%, and motilin 5.3% and 5.1%, respectively.

In each plasma sample, a protease inhibitor cocktail was added (SigmaFAST EDTA‐free; Sigma‐Aldrich, St. Louis, MO, USA), as well as 0.5 μmol/L DPP4 inhibitor (KR62432; Sigma‐Aldrich). Then, tubes were vortexed and centrifuged at 2500 RCF, 4°C, for 10 min to obtain plasma for analysis.

The gut peptide hormones GIP, active GLP‐1 (aGLP‐1), acyl‐ghrelin, and motilin were measured by multiplex electrochemiluminescence (MesoScale Discovery, Rockville, MD, USA). Appetite was measured by VAS (0–100) [29] for hunger, satiety, desire to eat, and prospective consumption at 10 min before meal‐taking and then 10, 60, 120, and 180 min following the meal.

While all 32 individuals with obesity who were included in the study underwent gastric motility recordings via SmartPill, hormone measurements were only available for 28 individuals.

### 2.2 Ethics

The Regional Ethics Board in Uppsala approved this study (no. 2010/184, date: 2010‐07‐21 and 2014‐03‐17). All participants gave written informed consent to participate in the study.

### 2.3 Statistical Analysis

All values are given as means ± standard error of the mean (SEM). The software Prism 10.0 (GraphPad, San Diego, CA, USA) was used for statistical analysis. Employing an exploratory approach for the limited biological aims of the study, biometric comparisons were conducted using the unpaired *t*‐test for continuous variables and the Chi‐square test for categorical variables. The Mann–Whitney *U*‐test was used to compare the GETs in healthy subjects and subjects with obesity. The Spearman rank correlation analysis was performed to evaluate the relationships between hormone concentrations and motility index, and to evaluate the relationship between hormone concentrations and VAS scores for hunger, satiety, desire to eat, and prospective consumption.

Logistic regression was carried out to assess the relationship between hormone concentrations and the gastric emptying process. The logistic regression models for gastric emptying used interpolation/extrapolation to estimate the hormone concentrations at different GET points. Interpolation/extrapolation was also used to assess hormone concentrations during the 60 min immediately preceding the emptying of the motility capsule from the stomach to the duodenum; the GET. Additionally, ANOVA with a linearity test was used to evaluate trends in hormone concentrations in the 60‐min window before the emptying of the motility capsule from the stomach to the duodenum.

## 3 Results

All SmartPill recordings with blood draws and appetite ratings were carried out without issues in the healthy controls. Among the people with obesity, five were omitted from the analysis of GET due to violating the study protocol and eating within 4 h of the standardized meal, while one was omitted due to gastroparesis. With respect to hormonal analyses, aGLP‐1 values from two healthy individuals were excluded due to assay errors.

### 3.1 Gastric Emptying

**FIGURE 1:**
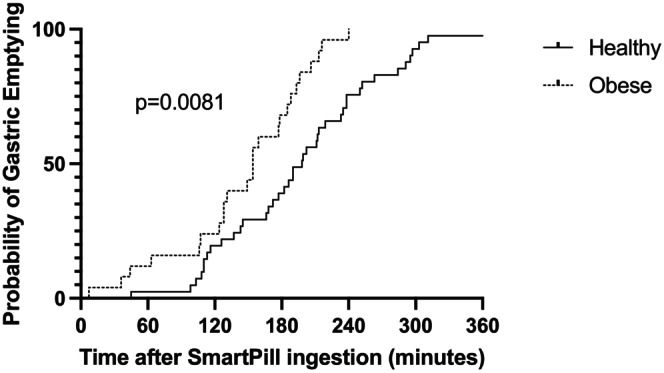
Gastric emptying time in healthy subjects versus people with obesity.

**FIGURE 2:**
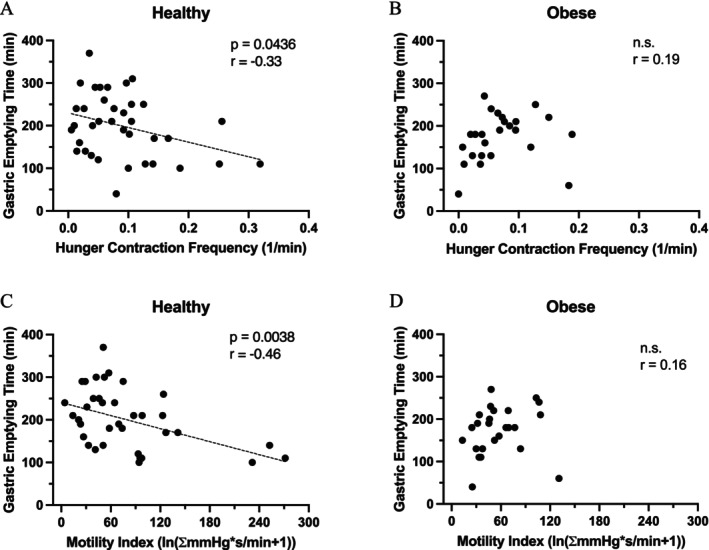
Upper panel: Hunger contraction frequency versus gastric emptying time in healthy subjects (A), and in people with obesity (B). Lower panel: Gastric motility index versus gastric emptying time in healthy individuals (C), and in people with obesity (D).

Gastric emptying rate was significantly more rapid in subjects with obesity as compared to healthy controls. The mean GET in subjects with obesity was 145 ± 12 min as compared to 198 ± 11 min in the healthy controls (*p* < 0.01) (Figure 1). In the control subjects, the GET was positively correlated with the gastric motility index (*p* < 0.01) and hunger contraction frequency (*p* < 0.05) (Figure 2A,C). Among people with obesity, GET showed no correlation with the gastric motility index or hunger contraction frequency before expulsion of the motility capsule over the pylorus (Figure 2B,D). In terms of appetite, no correlations were found between the hunger or satiety ratings and the GET.

### 3.2 Postprandial Gut Hormone Release and Gastric Motility Index

**FIGURE 3:**
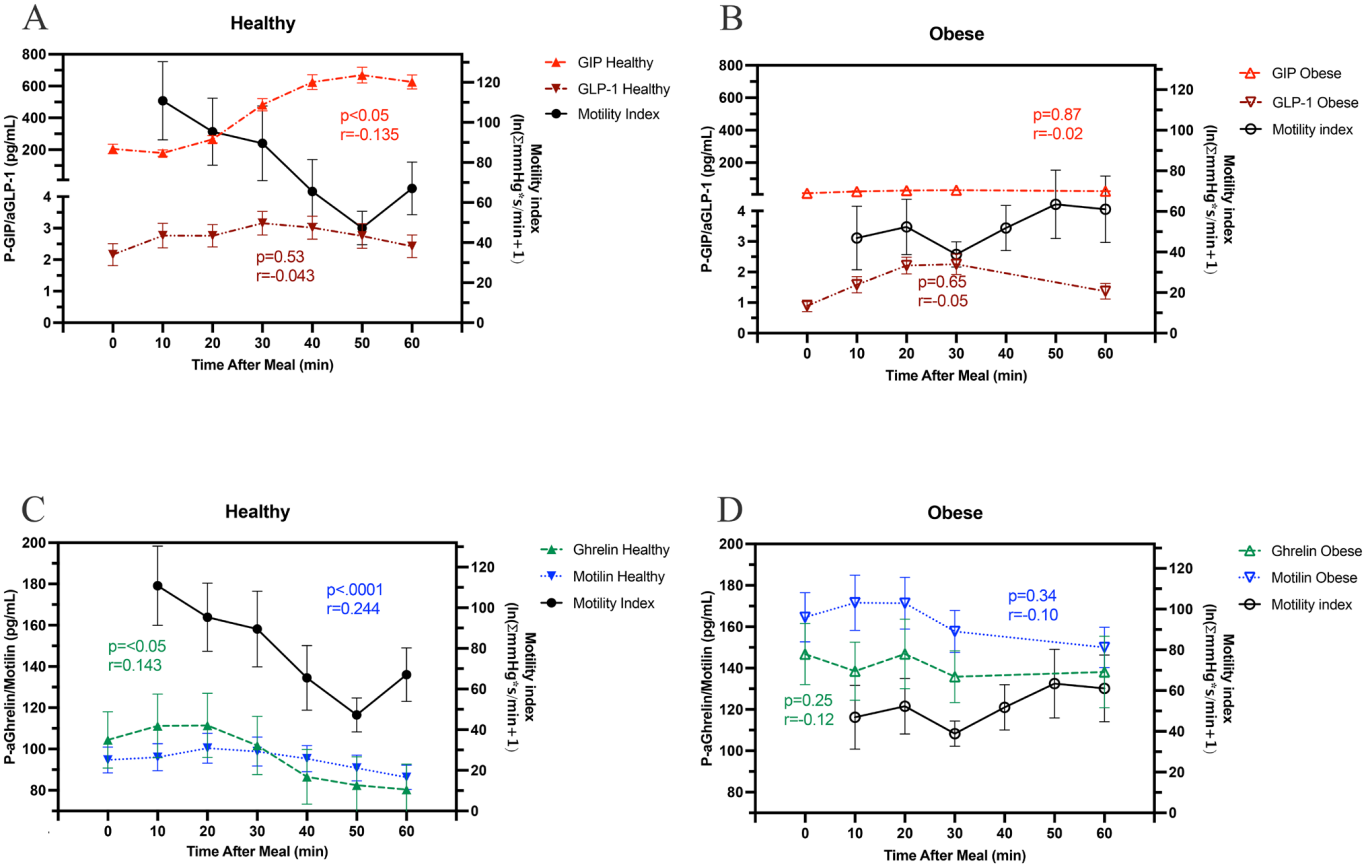
Upper panel: Postprandial GIP and GLP‐1 release and motility index in healthy subjects (A), and in people with obesity (B). Lower panel: Postprandial ghrelin and motilin release and motility index in healthy subjects (C), and in people with obesity (D).

In the control subjects, GIP rose following the meal, reaching a peak at 50 ± 3 min. The rise in GIP correlated with decreasing gastric motility index (*p* < 0.05), with its peak concentration of GIP coinciding with the trough in motility index at 50 min (Figure 3A). The concentration of active aGLP‐1 rose modestly following the meal, reaching a peak concentration at 34 ± 4 min, but was not correlated with the trough in motility index. The acyl‐ghrelin concentration began to decline approximately 20 min after meal intake to reach a nadir at 67 ± 7 min. These lowered acyl‐ghrelin concentrations also correlated with a decreased gastric motility index (*p* < 0.01). No discernible pattern in motilin secretion occurred following the meal, whereas higher plasma motilin concentrations strongly correlated with increased postprandial motility index (*p* < 0.0001) (Figure 3C).

Postprandial plasma GIP and aGLP‐1 levels were significantly lower in people with obesity as compared to healthy controls. The gastric motility index following the meal was considerably lower in people with obesity as compared to control subjects (*p* < 0.01) (Figure 3B). In people with obesity, GIP also rose postprandially but was not associated with any change in gastric motility index. Active GLP‐1 showed a slight rise following meal ingestion. Unlike in the healthy controls, acyl‐ghrelin concentration did not decline following meal consumption. No pattern in motilin secretion was appreciated after meal intake among people with obesity (Figure 3D).

In the control subjects, correlation analysis of the entire GET revealed a negative association between plasma GIP concentration and gastric motility index (*p* < 0.05). Active GLP‐1 was only numerically negatively associated with the motility index. Acyl‐ghrelin showed a positive association with the motility index (*p* < 0.01), as did motilin (*p* < 0.0001). (Table 1).

**TABLE 1:** Gut hormone concentration versus gastric motility index analyzed by Spearman rank correlation in healthy subjects, and in people with obesity.

Hormone	Healthy		Obesity	
	*R* value	*p* value	*R* value	*p* value
GIP	−0.12	0.02	−0.10	0.24
aGLP‐1	−0.09	0.09	−0.11	0.20
aGhrelin	0.15	< 0.01	0.04	0.62
Motilin	0.28	< 0.0001	−0.07	0.40

Abbreviations: aGhrelin, acyl‐Ghrelin; aGLP‐1, active glucagon‐like peptide‐1.

In people with obesity, aGLP‐1, GIP, acyl‐ghrelin, and motilin concentrations were not found to be significantly associated with the gastric motility index (Table 1).

### 3.3 Pre‐Emptying Gut Hormone Release and Gastric Motility

**FIGURE 4:**
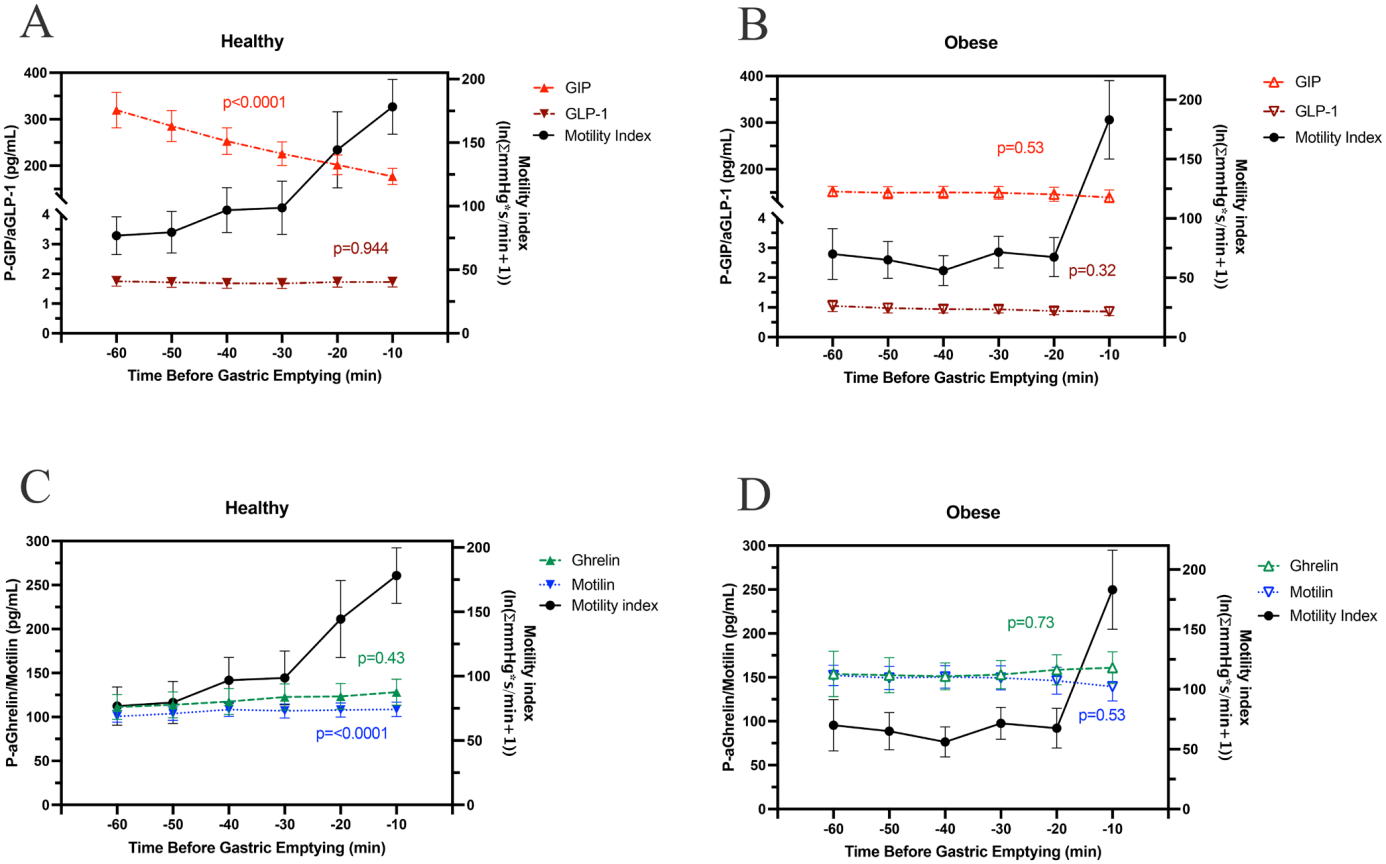
Upper panel: Gastric pre‐emptying GLP‐1 and GIP release and motility index in healthy subjects (A), and in persons with obesity (B). Lower panel: Gastric pre‐emptying ghrelin and motilin release and motility index in healthy subjects (C), and in people with obesity (D).

In the control subjects, gastric motility index steadily increased before emptying of the wireless motility capsule from the stomach (Figure 4A). The continuously rising motility index coincided with a statistically significant decrease in GIP (*p* = 0.005) and an increase in motilin (*p* < 0.0001) (Figure 4C). Logistic regression analysis revealed plasma concentrations of GIP (*p* < 0.0001) and aGLP‐1 (*p* < 0.05) to have an inverse relationship with the odds of gastric emptying. In contrast, concentrations of acyl‐ghrelin (*p* < 0.05) had a direct relationship with the odds of gastric emptying. Motilin concentration was not found to have a statistically significant relationship with the odds of gastric emptying (Table 2).

**TABLE 2:** Logistic regression analysis of gut hormone concentration versus gastric emptying among healthy subjects and people with obesity.

Hormone	Healthy		Obesity	
	Odds ratio (95% CI)	*p* value	Odds ratio (95% CI)	*p* value
GIP	0.991 (0.987–0.994)	< 0.0001	0.887 (0.827–0.939)	0.0002
aGLP‐1	0.696 (0.478–0.960)	0.04	0.535 (0.273–0.877)	0.035
aGhrelin	1.004 (1.001–1.007)	0.017	1.008 (1.002–1.016)	0.014
Motilin	1.007 (0.999–1.014)	0.075	0.996 (0.988–1.003)	0.238

Abbreviations: aGhrelin, acyl‐Ghrelin; aGLP‐1, active glucagon‐like peptide‐1.

In people with obesity, the pattern was different, with an abrupt increase in the gastric motility index immediately before the motility capsule emptying from the stomach entering into the duodenum (Figure 4B). At the same time, there were only numerical decreases of GIP, aGLP‐1, and motilin, along with an increase in acyl‐ghrelin leading up to the emptying of the motility capsule from the stomach in people with obesity, with no statistically significant linear trend appreciated with ANOVA analysis. Logistic regression analysis revealed concentrations of GIP (*p* < 0.01) and aGLP‐1 (*p* < 0.05) to have an inverse relationship with odds of gastric emptying, whereas acyl‐ghrelin (*p* < 0.05) had a direct relationship with the odds of gastric emptying. Motilin was not found to have a statistically significant relationship with odds of gastric emptying (Table 2).

### 3.4 Hunger

**FIGURE 5:**
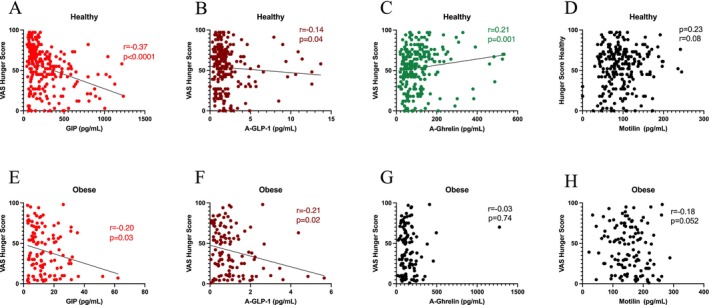
Gut hormone concentration versus hunger in healthy subjects and in people with obesity. Upper panel: GIP (A), GLP‐1 (B), ghrelin (C), and motilin (D) versus visual analog hunger score in healthy subjects. Lower panel: GIP (E), GLP‐1 (F), ghrelin (G), and motilin (H) versus visual analog hunger score in people with obesity.

In the control subjects, plasma concentrations of GIP (*p* < 0.0001) and aGLP‐1 (*p* < 0.05) were negatively correlated with hunger (Figure 5A,B). Acyl‐ghrelin had a positive correlation with hunger (*p* < 0.01) (Figure 5C), whereas motilin showed no such association (Figure 5D). In people with obesity, concentrations of GIP (*p* < 0.05) and aGLP‐1 (*p* < 0.05) were negatively correlated with hunger (Figure 5E,F). Acyl‐ghrelin and motilin concentrations showed no significant correlation with hunger (Figure 5G,H).

### 3.5 Satiety

**FIGURE 6:**
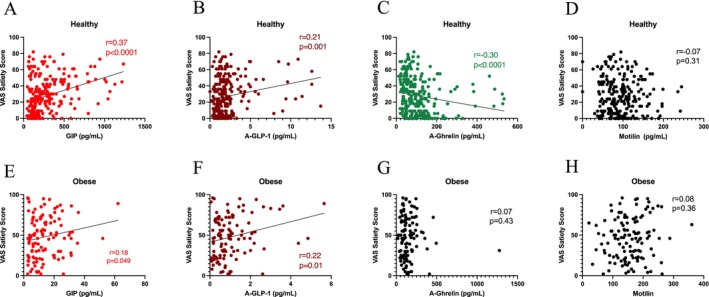
Gut hormone concentration versus satiety in healthy subjects and in people with obesity. Upper panel: GIP (A), GLP‐1 (B), ghrelin (C), and motilin (D) versus visual analog satiety scoring in healthy subjects. Lower panel: GIP (E), GLP‐1 (F), ghrelin (G), and motilin (H) versus visual analog satiety scoring in people with obesity.

In the controls, concentrations of GIP (*p* < 0.0001) and aGLP‐1 (*p* < 0.001) were positively correlated with satiety (Figure 6A,B). Acyl‐ghrelin was negatively correlated with satiety (*p* < 0.0001), while motilin had no association with satiety (Figure 6C,D). In people with obesity, concentrations of GIP (*p* < 0.05) and aGLP‐1 (*p* < 0.05) were positively correlated with satiety (Figure 6E,F). Motilin and acyl‐ghrelin showed no correlation with satiety (Figure 6G,H).

### 3.6 Desire to Eat

In healthy controls, concentrations of GIP (*p* < 0.0001) and aGLP‐1 (*p* < 0.05) were negatively correlated with desire to eat. Acyl‐ghrelin (*p* < 0.001) was positively correlated with the desire to eat, whereas motilin was not associated with desire to eat. In people with obesity, concentrations of GIP (*p* < 0.01) and aGLP‐1 (*p* < 0.01) were negatively associated with desire to eat, whereas motilin and acyl‐ghrelin had no association with desire to eat.

### 3.7 Prospective Consumption

In healthy controls, GIP was negatively associated with prospective consumption (*p* < 0.0001) while concentrations of acyl‐ghrelin (*p* < 0.05) and motilin (*p* < 0.05) were positively correlated with prospective consumption. Active GLP‐1 was not found to have a statistically significant association with prospective consumption. In people with obesity, GIP (*p* < 0.01) and aGLP‐1 (*p* < 0.01) were negatively associated with prospective consumption. No association was found between motilin or acyl‐ghrelin and prospective consumption.

## 4 Discussion

Our study discloses key differences in gastric motility, gut hormone release, and interactions between gut hormones and motility and appetite parameters between healthy individuals and individuals with obesity.

Our study shows differences in meal‐related gastric motility between healthy control subjects and people with obesity. Individuals with obesity had a more rapid GET than controls. Results of prior studies comparing GET in individuals with obesity and healthy individuals have been inconsistent [21, 22]. A more rapid GET raises the possibility of an abnormally rapid distribution of nutrients from the stomach with subsequent absorption in the small bowel leading to nutrient overload in individuals with obesity, as compared to lean control subjects. Studies have shown how more rapid gastric emptying leads to elevated postprandial glucose concentration in individuals with type 2 diabetes [30]. Even slight differences in gastric emptying may be necessary for nutrient absorption and the development of obesity over time.

Our monitoring of the gastric emptying process with the wireless motility capsule and plasma gut hormone concentrations discloses key differences in persons with obesity and lean healthy controls. In healthy controls, there was an apparent rise in the concentration of incretins GIP and GLP‐1 following the meal, which coincided with a decrease in gastric motility index. As concentrations of GIP and GLP‐1 then declined again, gastric emptying was more likely to occur. However, in subjects with obesity, the release of these hormones was blunted, and their relationship with gastric emptying was distorted. Hence, the rapid emptying in people with obesity may be a consequence of differential gut hormone signaling, with lower plasma levels of GIP and GLP‐1 leading to rapid emptying in those with obesity compared to lean controls. To our knowledge, this is the first study in humans that has shown a negative correlation between GIP concentration and gastric motility. Previously, peripheral infusions of 2–5 pmol/kg/min of GIP were not effective in slowing gastric emptying nor affecting appetite [26]. However, these doses were likely too low to see an effect. Furthermore, studies in humans show GLP‐1 to be an inhibitor of gastric motility and emptying [24, 25], seemingly with a higher potency compared with GIP [26], but with a similar effect on absorption as shown for glucose [31]. In line with this, we found higher plasma levels of GIP and GLP‐1 to be associated with lower odds of gastric emptying in both healthy controls and persons with obesity, but less apparent and at lower hormone levels in subjects with obesity.

With respect to the counter‐regulatory mechanisms of the anti‐incretins ghrelin and motilin for gastric emptying, we found higher plasma levels of ghrelin to be associated with increased odds of gastric emptying. However, healthy controls and people with obesity displayed different patterns of ghrelin secretion. In healthy individuals, ghrelin levels decreased immediately after the meal before increasing along with the motility index before the expulsion of the motility capsule from the stomach. People with obesity displayed a different pattern with no suppression of ghrelin secretion after food intake, and subsequently plasma levels of ghrelin began to rise earlier than in the healthy controls, which may play a role in the earlier gastric emptying observed in individuals with obesity. The response of motilin to food intake showed a similar pattern to that of ghrelin.

It is plausible that the hastened gastric emptying in people with obesity could be explained by lower concentrations of GIP and GLP‐1, leading to less suppression of gastric motility, in combination with an earlier rise of ghrelin and motilin, driving gastric emptying with increased availability of nutrients for absorption in the small intestine.

In healthy control subjects, GIP negatively correlated with the gastric motility index. This agrees with the fact that GIP does not induce food intake [32], but rather promotes a negative energy balance [11, 33]. Hence, novel GLP‐1/GIP hybrid peptides have been shown to lead to marked weight loss in preclinical and clinical settings [34, 35]. The therapeutic efficacy of this combination surpasses that of GLP‐1 agonists alone, suggesting that GIP receptor agonism exerts additional weight‐reducing effects in a pharmacological context [36].

An unexpected finding in our study was that GLP‐1 showed no significant association with the gastric motility index. This may be because the meal size was too small to elicit a robust GLP‐1 response. In addition, the majority of GLP‐1‐secreting cells are found in the lower GI tract, meaning that a GLP‐1 response would not be expected until later after the meal. Similarly, accelerated intestinal transit, for example, by gastric bypass, provides rapid release of GLP‐1 [37], indicating release from lower parts of the gut at a later time. Still, GLP‐1 is an essential player in suppressing gastric motility and increasing satiety [38], as shown repeatedly in clinical trials.

Our results raise the question of whether endocrine resistance in the control of gastrointestinal motility could be present in people with obesity. In our healthy controls, ghrelin and motilin showed parallel associations with the gastric motility index, while the hunger contraction frequency and gastric motility index correlated with GET. In persons with obesity, neither gastric motility index nor hunger contraction frequency was correlated with GET. While the gastric motility index steadily decreased after food intake in the healthy controls, a similar decrease did not occur in people with obesity. Before the emptying of the motility capsule from the stomach, gastric motility index increased steadily in the healthy controls, but not in people with obesity; there was an abrupt increase in motility index only immediately prior to emptying. While the plasma levels of GIP, ghrelin, and motilin were found to correlate with gastric motility index in the healthy controls, none of these correlated with gastric motility index in people with obesity. To this end, gastric phase III motor activity and plasma peaks of motilin occur less frequently in patients with severe obesity [39]. Together with our present data, a disruption of the prandial endocrine regulation of gastric emptying may be at hand in subjects with obesity.

Our study shows both GIP and GLP‐1 to be strongly positively correlated with satiety and negatively correlated with hunger. These findings support the hypothesis that GIP, in addition to GLP‐1, has a vital role in regulating appetite in vivo. Interestingly, ghrelin was positively correlated with hunger and negatively correlated with satiety in healthy individuals but did not correlate with hunger or satiety in people with obesity. This finding points to ghrelin resistance, which has been previously discussed as a key characteristic of obesity [40]. It has been postulated that ghrelin sensitivity decreases in subjects with obesity to maintain the higher weight that develops in times of food abundance and then rebounds in times of limited food availability, such as, diet‐induced weight loss, to maintain homeostasis [41]. Even though motilin and ghrelin share considerable structural similarities, we did not find motilin to correlate with hunger or satiety in neither healthy controls nor in people with obesity, suggesting ghrelin may be more relevant for control of gastric functions. In contrast, motilin has a typical role in the initiation of interdigestive phase III of the migrating motor complex [42].

## 5 Limitations

One limitation of our study is that we had no weight adjustment with respect to the size of the meal delivered to the healthy individuals and persons with obesity. This is of importance for the release and effects of GLP‐1, where we today have broad knowledge of its attenuated meal response in obesity [2]. In spite of this, we were able to show clear physiological meal responses for most participants, while some were hampered with a low release amplitude. Furthermore, people with obesity were older than the healthy controls, meaning that age could be a possible confounding variable of our results.

A deviation in our study was that blood samples in the healthy subjects were also drawn at 40 and 50 min into the meal, while in the group with obesity blood samples were not drawn at those time points. Given that the timing of blood draws was not carried out according to the temporal profile of the gastric emptying process, interpolation of these data points was carried out to estimate the hormone concentrations at those time points.

To this end, GET was assessed using a wireless motility capsule, while gastric emptying is classically assessed using gastric scintigraphy. Nevertheless, the wireless motility capsule has been shown to agree with gastric scintigraphy [43] and even surpass the accuracy of scintigraphy in the diagnosis of gastroparesis [44]. Furthermore, the use of the wireless motility capsule was critical to the study design as it enabled analysis of the association between various gut hormone concentrations, hunger and satiety, as well as the desire to eat and prospective consumption, hunger contractions, and gastric motility indices at discrete times; an analysis that would not have been possible with scintigraphy. Lastly, an essential limitation of this study is its exploratory observational design, precluding interpretation of findings as causal.

## 6 Conclusions

People with obesity demonstrate clear differences to healthy controls with respect to gastric emptying, gastric motility index, and endocrine regulation of appetite during the meal‐induced gastric filling and emptying cycle. GIP may play an important role in suppressing appetite and motility, similar to the well‐known effects of GLP‐1. The hormonal cycling with an earlier rise of ghrelin among people with obesity, coupled with lower levels of suppression from GIP and GLP‐1, may underlie the more rapid gastric emptying in people with obesity, leading to shorter meal intervals as a mechanism for over‐eating behavior. Furthermore, compared to healthy controls, data from people with obesity indicate ghrelin resistance with no apparent suppression of ghrelin release after a meal, and in line with this, the absence of correlation between plasma ghrelin concentrations and hunger or satiety signaling.

## Author Contributions

D.J. and P.M.H. were responsible for the design of the study and the protocol, and the first draft of the manuscript. H.O.D., K.E., and P.M.H. conducted the study. D.J., D.L.W., H.O.D., M.U., and K.E. extracted and analyzed data. D.J., D.L.W., M.S., and P.M.H. interpreted results and reviewed the final manuscript.

## Conflicts of Interest

The authors declare no conflicts of interest.

## Data Availability

The data that support the findings of this study are available from the corresponding author upon reasonable request.
